# Expression and *In Silico* Analysis of the Recombinant Bovine Papillomavirus E6 Protein as a Model for Viral Oncoproteins Studies

**DOI:** 10.1155/2013/421398

**Published:** 2013-06-26

**Authors:** J. Mazzuchelli-de-Souza, R. F. Carvalho, R. M. Ruiz, T. C. Melo, R. P. Araldi, E. Carvalho, C. E. Thompson, M. P. Sircili, W. Beçak, R. C. Stocco

**Affiliations:** ^1^Laboratório de Genética, Instituto Butantan, Secretaria de Estado da Saúde, Avenida Vital Brasil, 1500 Butantã, 05503-900 São Paulo, SP, Brazil; ^2^Programa de Pós-graduação Interunidades em Biotecnologia, Instituto de Ciências Biomédicas, Edifício ICB-III-Cidade Universitária, Universidade de São Paulo, Avenida Prof. Lineu Prestes, 2415 Butantã, 05508-900 São Paulo, SP, Brazil; ^3^Programa de Pós-graduação em Biologia Estrutural e Funcional, Universidade Federal de São Paulo, Rua Botucatu 740, Vila Clementina, 04023-900 São Paulo, SP, Brazil; ^4^Laboratório de Biotecnologia Molecular, Instituto Butantan, Secretaria de Estado da Saúde, Avenida Vital Brasil 1500, Butantã, 05503-900 São Paulo, SP, Brazil; ^5^Laboratório Nacional de Computação Científica, Avenida Getúlio Vargas 333, Quitandinha, 25651-075 Petrópolis, RJ, Brazil; ^6^Departamento de Biologia, Universidade Federal da Integração Latino-Americana (UNILA), Avenida Tancredo Neves 6731 bloco 4, 85867-970 Foz do Iguaçú, PR, Brazil

## Abstract

Bovine papillomaviruses (BPVs) are recognized as the causal agents of economical relevant diseases in cattle, associated with the development of tumors in skin and mucosa. The oncogenesis process is mainly associated with different viral oncoprotein expressions, which are involved in cell transformation. The expression and characterization of recombinant viral oncoproteins represent an attractive strategy to obtain biotechnological products as antibodies and potential vaccines, Thus, the aim of this work was to clone and express the BPV-1 and BPV-2 E6 recombinant proteins and perform *in silico* analysis in order to develop a strategy for the systematic study of other papillomaviruses oncoproteins. The results demonstrated that BPV-1 and BPV-2 E6 recombinant proteins were expressed and purified from bacterial system as well as its *in silico* analysis was performed in order to explore and predict biological characteristics of these proteins.

## 1. Introduction

Different papillomaviruses (PVs) have been described as infectious agents of the vertebrates species, including domestic animals and human beings [1, 2]. The correlation between the papillomavirus infection and the cellular malignant progression is associated with the expression of viral oncoproteins. These proteins act on different aspects of the tumoral suppression cascades as well as on the ones that take part in the control of cell cycle and immune response. Viral oncoproteins can also interact with cellular DNA. Altogether, these actions can induce mutational changes in the host cell chromatin [3].

Currently, the *Papillomaviridae* family is divided into 16 genera according to their genomic organization [4, 5]. These small (55–60 nm), nonenveloped viruses have a genome of a double-stranded circular DNA molecule of approximately eight kilobases [6], codifying functional, early (E) proteins, and structural, late (L) proteins, expressed at different stages of the viral cycle. With at least eight potential open reading frames, the viral genome also consists of a noncoding region, the long control region (LCR), associated with the viral transcriptional regulation. The E region encodes the replication and transcription regulatory proteins E1, E2, and the transforming proteins E5, E6, and E7, which are associated with uncontrolled cell proliferation and differentiation [7]. The E4 protein formed by alternative splicing of genes E1 and E1/E4 transcripts (E1–E4) is associated with the release of the virions through the disruption of the cytoskeleton structure [8–10]. It is also shown that actin cytoskeleton was altered in BPV-1 E6-transformed cells through E6 interaction with the focal adhesion protein paxillin [11]. On the other hand, the L region encodes structural proteins L1 and L2 that assemble into the capsid during the viral particle maturation [12]. Specifically, L1 is the most conserved gene within PV genome and has therefore been used for the identification of new PVs types [4]. 

The bovine papillomavirus (BPV) is recognized as the causal agent of benign and malignant tumors in cattle, such as cutaneous papillomas, urinary bladder, and esophagus cancer. This virus is distributed worldwide, being associated with severe economic losses in meat, milk, and leather production. Thirteen types of the BPVs are currently well characterized and classified into three distinct genera, Delta, Epsilon, and Xi, and have been characterized and associated with different histopathological lesions [13]. Specifically, the BPV-1 and 2 are classified as *Deltapapillomaviruses* [14, 15]. Characteristically, these types induce the appearance of fibropapillomas, associated with the recruitment of the subepithelial fibroblasts [16] and have the ability to infect different host species, not only bovines, causing the equine sarcoid [15]. Lately, the genome of a new Delta-BPV type (BPV-13) was fully sequenced [17].

The BPV-1 is commonly associated with lesions in the teats and udder [13, 18, 19]. BPV-1 can cause fibropapillomas of the penis, leading to necrosis and the loss of reproductive function [20]. BPV-2 is the causal agent of malignant tumors in the bladder [21]. Both types have also already been detected in peripheral blood and in tissues of the reproductive tract, and their vertical transmission has been suggested [16, 21–26].

The first evidence of the oncogenic properties of E6 protein came from studies on human tumors cell lineages derivate from uterine cervix where E6 was found expressed and maintained many years after the initial transformation events [27–29]. The E6 and E7 gene products are essential in the process of cell transformation and immortalization [28, 30]. Particularly, E6 protein has a central role as a carcinogen factor because it binds to p53, a major tumor suppressor protein, inducing its degradation [4]. Studies conducted with different cervical cancer cell lines infected with HPV-16 showed that the only expressed viral proteins were E6 and E7, leading to the speculation that they could be expressed like fusion proteins, an important indicator for the malignant progression [31]. It is also suggested that the genes E6 and E7 have a synergic action during the induction of genital human keratinocytes immortalization, although in some other cell types, like mammary epithelial cells, they may act separately [32].

Knowing the importance of E6 protein, the aim of this work was to clone and express the BPV-1 and BPV-2 E6 recombinant proteins enabling the development of antibodies and vaccines and to perform *in silico* analysis in order to develop a strategy for the systematic study of other papillomaviruses oncoproteins.

## 2. Material and Methods

### 2.1. E6-1 and E6-2 Gene Amplification

The following specific primers were designed: E6-1 sense primer, 5′-GAAAACCTGTATTTTCAGGGCTAGGACCTGAAACCTTTTGC-3′; and E6-1 antisense primer, 5′-GGC*CTCGAG*CTGCAGGTGAATCATCCAAG-3′, E6-2 sense primer, 5′-GAAAACCTGTATTTTCAGGGCATGGACCTGCAAAGTTTTTC-3′; and E6-2 antisense primer, 5′-GGC*CTCGAG*GAATCATCCAAGTTTCTA-3′. Underlined letters indicate the nucleotides for TEV protease site, and italic underlined letters indicate *Xho*I restriction site. The primers were designed based on complete genome sequences deposited in GenBank (accession numbers X02346 and M20219.1). These primers were used in a PCR reaction to amplify E6 gene, using the genomic DNA of BPV-1 or BPV-2 previously cloned in pAT153 vector as a template [33]. A Palm- Corbett Cycler Cobbert Research Version 2.1.7 (Uniscience) was used with the following amplification program: an initial denaturation step at 95°C for 4 min followed by 30 cycles at 95°C for 1 min, 50°C for 30 sec, 72°C for 1 min, and finally, 5 min elongation step at 72°C.

### 2.2. Cloning and Subcloning

The amplified PCR products were detected in a 1% agarose gel electrophoresis, excised from the gel, and purified with Invisorb Fragment Clean Up Kit (Invitek). The purified amplicons were cloned in pCR4-TOPO vector (Invitrogen). The resulting constructs were cloned in transformed *E. coli* DH5a competent cells, and positive clones were selected from plates supplemented with ampicillin. Plasmid DNA was prepared from overnight grown cultures with a WIZARD Mini Prep Purification Kit (Promega) following the manufacturer's recommendations. Plasmids were digested with *Eco*RI and *Xho*I to check the insert presence. Purified inserts were subcloned into the pET-28(+) vector (Merck), which was previously digested with the same enzymes. T4 DNA ligase (Invitrogen) was used for the ligation reaction. Recombinants pET-E61 and pET-E62 were then used to transform *E. coli* BL21 (DE3) competent cells cells by heat shock. Positive recombinant clones were selected on LB plates containing kanamycin, and the correct insertion of the E6 ORF into the cloning sites was verified by DNA sequencing.

### 2.3. Protein Expression

Transformed *E. coli* BL21 (DE) cells harboring the correct expression construct (pET-E61 and pET-E62) were grown in 1L LB broth containing kanamycin (50 *μ*g/mL) at 37°C until the growth reached log phase (OD600 = 0.6). IPTG at a final concentration of 1 mM was added to the cultures to induce recombinant protein expression. Induced bacterial cultures were pelleted by centrifugation at 10,000 g for 30 min at 4°C.

### 2.4. Electron Microscopy

Samples of transformed *E. coli* BL21 cultured cells with the recombinant plasmid E6-1/pET-28a and with the empty pET-28a vector (negative control) were induced for recombinant protein expression. Fractions of these cultures were centrifuged and resuspended in approximately 1.0 mL of glutaraldehyde. These samples were sent to the Department of Cell Biology and Development at the Institute of Biomedical Sciences-ICB II to be prepared for electron microscopy (ME) for the verification of the E6 recombinant expression through the observation of inclusion bodies. ME observations and image recording were performed at the Laboratory of Cellular Biology, Instituto Butantan, using a transmission electron microscope LEO 906E.*2.5*.

### 2.5. Protein Purification

In order to purify the E6 recombinant proteins, the bacterial pellets were resuspended in 20 mM TrisHCl, NaCl 500 mM, and pH 8.0, supplemented with protease inhibitor (PMSF at a final concentration of 2 mM), and lysed with a French Press. The cell lysates were centrifuged at 6000 g for 60 min at 4°C. The pellets were resuspended in 20 mM TrisHCl, 500 mM NaCl, 8 M ureia, 50 mM imidazole, pH 8.0, and final volume 25 mL. Aliquots of the clarified suspension were collected and analyzed in 17% SDS-PAGE. These tagged recombinant proteins were purified by Ni Sepharose 6 Fast Flow (GE Health Care): the clarified supernatants were passed through columns of charged resin and subsequently washed with 20 mM Tris-HCl, pH 8.0, 500 mM NaCl, 6 M urea, and 50 mM imidazole. The recombinant proteins were then eluted with 500 mM imidazole in the same buffer. The proteins quantification was determined by the Bradford method [34]. Purified recombinant proteins were separated by 17% SDS-PAGE and transferred to nitrocellulose membranes. Membranes were blocked with 5% powdered nonfat dry milk in phosphate buffered saline containing 0.05% Tween 20 (PBST) and incubated for 2 h using anti-his monoclonal antibody (GE) (1 : 3000). Blots were then submitted to three 5 min washes with TBST and incubated with Goat Anti-Mouse IgG Horseradish Peroxidase (1 : 2000) for 1 h at room temperature. Bands were visualized by chemiluminescence reaction in the presence of H_2_O_2_ and using diaminobenzidine (DAB). The reaction was stopped with distilled water.

### 2.6. Bioinformatics

The nucleotide sequences generated by sequencing of E6-1 and E6-2 cloned genes were translated into amino acids with the BioEdit 7.1.3.0 [35]. The obtained nucleotide and amino acids sequences were aligned with sequences deposited in Nucleotide Collection and Protein Data Bank using BLASTN and BLASTP algorithms (http://blast.ncbi.nlm.nih.gov/Blast.cgi). Sequence alignments were also performed using the BioEdit software, and the identity matrix was calculated. The topology diagrams of the recombinant proteins was generated with the PDBsum software [36].

The analysis of conserved regions of the proteins E6-1 to E6-2 recombinants were performed by comparing all E6 protein sequences of other PVs already deposited using ConSurf server [37]. The degree of conservation for each amino acid was pointed out in a linear sequence. 

In addition, the analysis of the antigenicity properties was performed using the JaMBW Edition 1.1 software [38].

## 3. Results and Discussion

### 3.1. E6-1 and E6-2 Gene Amplification and Cloning

E6-1 and E6-2 gene PCR products showed bands in the gel with approximately 500pb. E6-1/TOPO and E6-2/TOPO were successfully cloned in *E. coli* DH5a competent cells as indicated with double digestion of the recombinant TOPO vectors. Subsequently, E6-1/pET and E6-2/pET were subcloned in *E. coli *BL21 competent cells as indicated by double digestion and sequencing. DNA sequencing showed that the cloned genes were inserted in the correct frame of pET-28a (+). The primers sets were also effective for DNA sequencing.

### 3.2. Electron Microscopy

**Figure 1:**
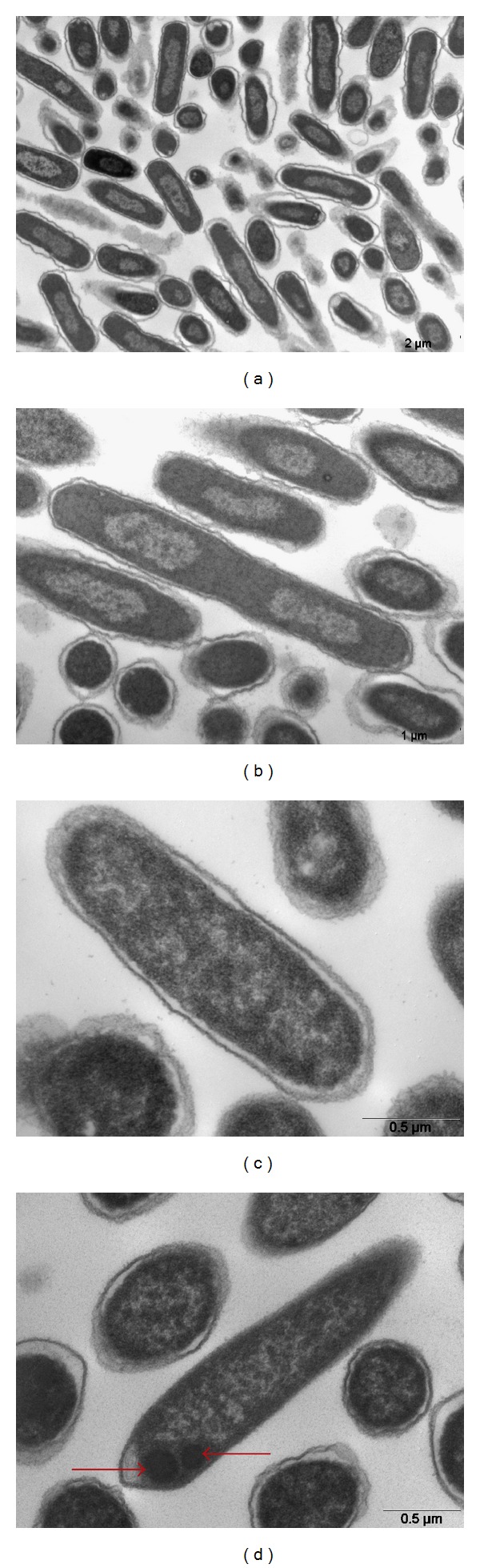
Electron microscopy *E. coli* BL21 (DE) not induced and induced by IPTG. (a) Transformed bacteria—plasmid pET-28a, without E6-1 not induced, 12.930X; (b) transformed bacteria—plasmid pET-28a, without E6-1 induced, 16.700X; (c) transformed bacteria—plasmid pET-28a, with E6-1 not induced, 35.970X; (d) transformed bacteria—plasmid pET-28a, with E6-1 induced 27.800X. Arrows: inclusion bodies. Transmission Microscopy LEO 906E, Laboratório de Biologia Celular do Instituto Butantan.

In regard to electronic microscopy (EM), both induced and noninduced cultured bacteria used as negative control (transformed with pET-28a, but without E6 insert) showed no inclusion corpuscles (Figures 1(a) and 1(b)). On the other hand, EM of the cultured, induced *E. coli* BL21 cells transformed with E6-1/pET-28a revealed the presence of inclusion bodies (Figure 1(d)), suggesting the presence of recombinant protein expression. As before, these were not observed in noninduced E6-1/pET-28a bacteria (Figure 1(c)). Papillomavirus E6 proteins are notoriously difficult to express and purify and unfused E6 proteins form insoluble aggregates upon bacterial overexpression [39]. However, in this study, the feasibility of E6-1 and E6-2 purifications from a bacterial expression system was demonstrated.

### 3.3. E6 Recombinant Protein Expression and Purification

Cloning and expression of different papillomavirus oncoproteins in bacterial vectors have already been done, enabling structural studies [40]. In the present work, the E6 gene of both BPV-1 and BPV-2 was cloned in a bacterial expression system, with the respective recombinant proteins being purified. 

**Figure 2:**
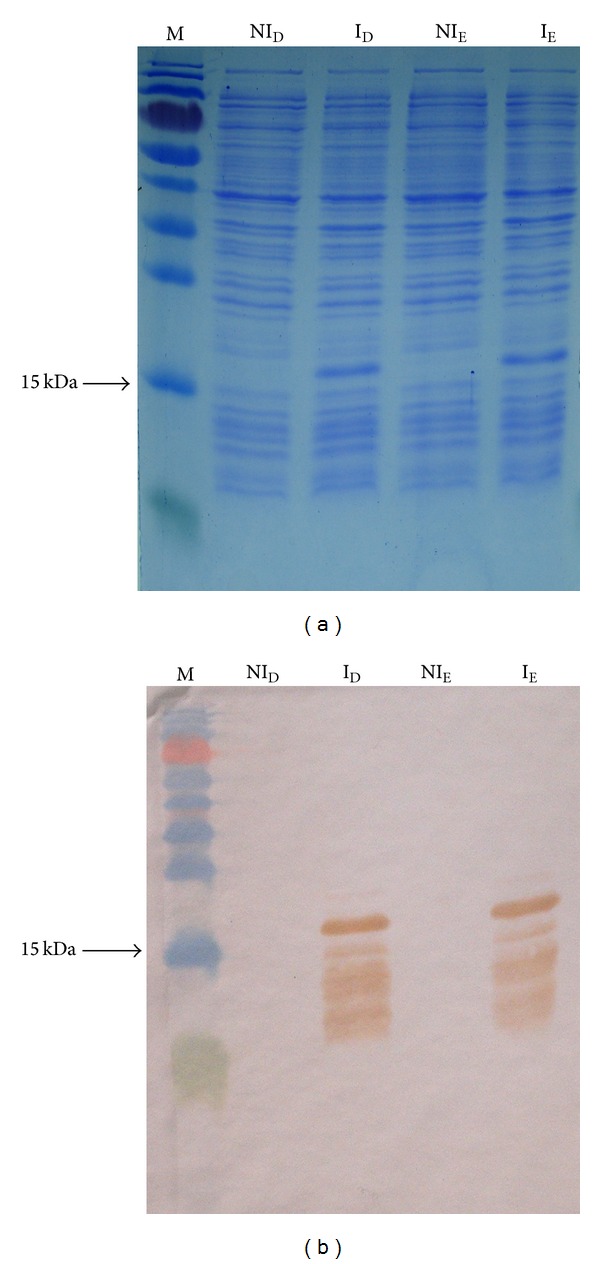
(a) Induction of E6-1 recombinant protein. M: molecular weight marker; NI_D_: noninduced D colony; I_D_: induced D colony; NI_E_: noninduced E colony; I_E_: induced E colony. SDS-PAGE 17% stained with Coomassie blue. (b)Western blotting of E6-1 recombinant protein induction. M: molecular weight marker; NI_D_: noninduced D colony; I_D_: induced D colony; NI_E_: noninduced E colony; I_E_: induced E colony.

SDS-PAGE and Western blotting analysis using an anti-his tag antibody demonstrated that the large majority of detected fusion proteins migrated predominantly as a single band with an approximate expected molecular mass of 16 kDa (Figure 2(a)). However, Western blotting showed other bands also, indicating the possible occurrence of protein dimerization (Figure 2(b)). The purified recombinant eluted proteins were also examined by SDS-PAGE and Western blotting as before, with observed bands being approximately 16 kDa. 

### 3.4. Bioinformatics Analysis

#### 3.4.1. Alignment and Identity Matrix

The identity matrix showed 0.99 of similarity between E6-1 recombinant and reference (X02346) sequences of nucleotides. The amino acid sequence of the E6-1 recombinant protein showed 0.99 of identity with PDB codes 3PY7 sequence which has been considered as the protein sequence reference.

It was observed that, when translated into amino acids, two mutations (A78G and T48C) were silent, that is, no change in the amino acids was generated. The other two mutations (A73T and T155C) generated different amino acids (Table 1). 

Differences between recombinant cloned E6-2 and deposited corresponding sequences were also observed. The identity matrix showed 0.99 of similarity between E6-2 recombinant and reference (M20219.1) sequences of nucleotides. Amino acid sequence of the E6-2 recombinant protein showed 0.98 of identity with UniProtKB/Swiss-Prot codes P11302.1 sequence which has been considered as the protein sequence reference. It was observed that, when translated into amino acids, all three mutations (T68C, T45G, and A405C) generated different amino acids (Table 2). 

#### 3.4.2. Topology

**Figure 3:**
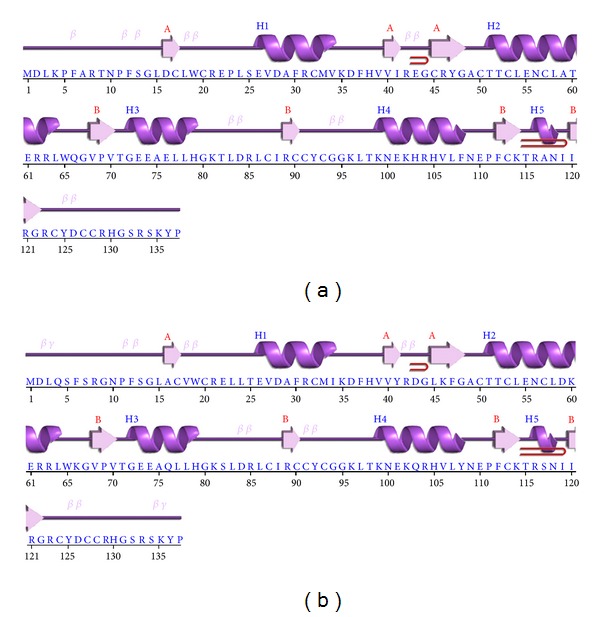
Topology diagram recombinants E6-1 and E6-2, respective sequences (a) E6-1 recombinant; (b) E6-2 recombinant.

Topology diagram of recombinant proteins, E6-1 and E6-1 was generated. The presence of seven *β*-sheets and five *α*-helices was observed in both diagrams (Figures 3(a) and 3(b)).

#### 3.4.3. Antigenicity Prediction

**Figure 4:**
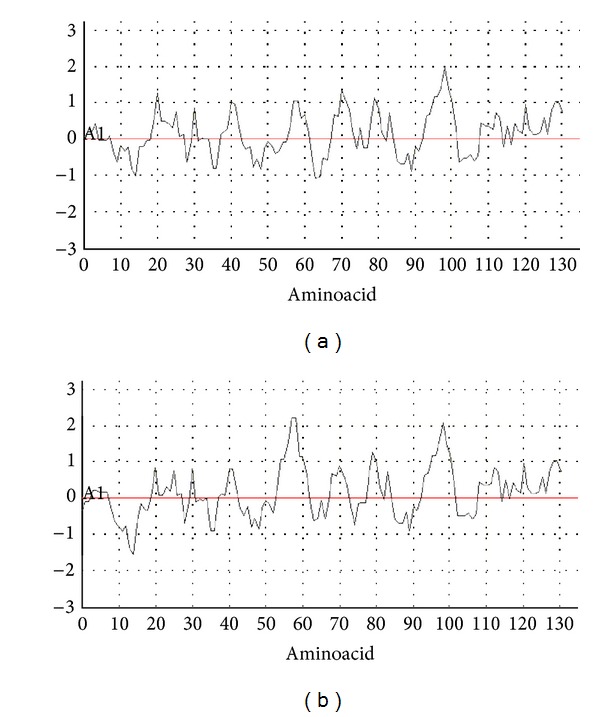
Antigenicity-recombinants E6-1 and E6-2, (a) E6-1 recombinant; (b) E6-2 recombinant.

According to the antigenicity graph, E6-1 recombinant protein sequence showed one peak near amino acids 90 and 100 (CCYCGGKLTKNEKHR), and E6-2 recombinant protein showed two peaks near amino acids 50 and 60 (CTTCLENCLDKE), and amino acids 90 and 100 (CCYCGGKLTKNEKQR) were predicted as especially immunogenic (Figures 4(a) and 4(b)). Interestingly, these potential immunogenic, mapped regions could represent targets for the development of new designed antibodies.

#### 3.4.4. Conserved Regions

**Figure 5:**
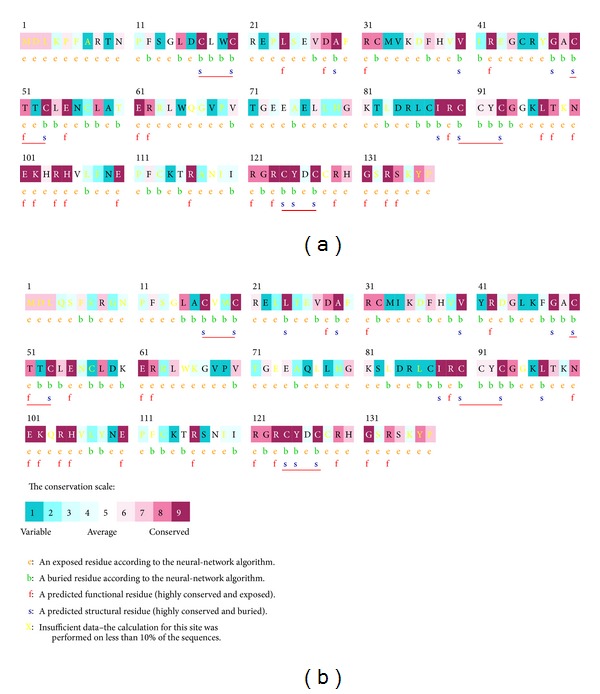
Protein sequences E6-1 e E6-2 conservation levels. (a) E6-1 recombinant; (b) E6-2 recombinant. CXXC red.

*In silico* prediction identified conserved regions between E6-1 to E6-2 proteins as well as from other papillomaviruses species. CXXC motifs were localized at regions associated with the binding of zinc atoms (Figures 5(a) and 5(b)). 

Usually, papillomavirus E6 proteins share a common architecture consisting of two zinc-binding domains (E6-N and E6-C). These structural features indicated that E6 can interact directly with DNA molecule, acting as a transcriptional activator [41–43]. Both BPV-1 and BPV-2 encode an E6 protein of 137 amino acids that acts as a transcriptional activator, p53 and paxillin ligand, presenting also telomerase activity. Here, the primary amino acid sequences of these recombinant proteins were analyzed *in silico* for comparison with virtual protein sequences deposited in GenBank. The presence of divergences which may represent functional differences were observed. It is emphasized that the DNA sequencing in our laboratory was redundant in order to cover the entire E6 gene sequence for at least three times. 

Among papillomaviruses oncoproteins, conserved regions were maintained in regard to the structure and function of these proteins. For example, E7 protein has 127 amino acids and a zinc finger domain [44]. Recombinant E7 protein with mutated regions showed lower efficiency in transforming activity [45]. On the other hand, several studies indicated that the hydrophobic nature of the BPV E5 protein has a crucial importance in conferring the transforming activity [46]. These essential amino acid residues are highly conserved among papillomaviruses as previously reported [47].

## 4. Conclusions

The cloning and recombinant protein expression of E6-1 and E6-2 in bacterial system proved to be a feasible methodological approach. For the first time, BPV-2 E6 protein is expressed and purified in a bacterial system. The purification of E6 BPV recombinant protein as well its structural and antigenicity analyses could allow the production of biotechnology material such as antibodies and vaccines candidates. This work could be also employed as a model for the obtainment of other papillomaviruses recombinant oncoproteins.

## Figures and Tables

**Table 1:** Mutations in nucleotide and aminoacid sequences of BPV1 E6 (bold).

E6 BPV-1										
Nucleotide				Codon			Amino acid			
Position	Ref.		Rec.	Ref.		Rec.	Ref.		Rec.	Position
48	T	→	C	GA**U**	→	GA**C**	D	=	D	16
73	A	→	T	**A**CA	→	**U**CA	T	≠	**S**	25
78	A	→	G	GA**A**	→	GA**G**	C	=	C	26
155	T	→	C	A**U**U	→	A**C**U	I	≠	**T**	52

Ref.: reference sequences (accession number X02346 and PDB codes 3PY7); Rec.: recombinant sequences obtained in this study.

**Table 2:** Mutations in nucleotide and aminoacid sequences of BPV2 E6 (bold).

E6 BPV-2										
Nucleotide				Codon			Amino acid			
Position	Ref.		Rec.	Ref.		Rec.	Ref.		Rec.	Position
68	C	→	T	C**C**U	→	C**U**U	P	≠	**L**	23
134	G	→	T	**G**UG	→	**U**UG	V	≠	**L**	45
405	C	→	A	AA**C**	→	AA**A**	N	≠	**K**	135

Ref.: reference sequences (accession number M20219.1 and UniProtKB/Swiss-Prot codes P11302.1); Rec.: recombinant sequences obtained in this study.
